# Detection of Atherosclerotic Inflammation by ^68^Ga-DOTATATE PET Compared to [^18^F]FDG PET Imaging

**DOI:** 10.1016/j.jacc.2017.01.060

**Published:** 2017-04-11

**Authors:** Jason M. Tarkin, Francis R. Joshi, Nicholas R. Evans, Mohammed M. Chowdhury, Nichola L. Figg, Aarti V. Shah, Lakshi T. Starks, Abel Martin-Garrido, Roido Manavaki, Emma Yu, Rhoda E. Kuc, Luigi Grassi, Roman Kreuzhuber, Myrto A. Kostadima, Mattia Frontini, Peter J. Kirkpatrick, Patrick A. Coughlin, Deepa Gopalan, Tim D. Fryer, John R. Buscombe, Ashley M. Groves, Willem H. Ouwehand, Martin R. Bennett, Elizabeth A. Warburton, Anthony P. Davenport, James H.F. Rudd

**Affiliations:** a Division of Cardiovascular Medicine, University of Cambridge, Cambridge, United Kingdom; b Heart Center, Rigshospitalet, Copenhagen, Denmark; c Department of Clinical Neurosciences, University of Cambridge, Cambridge, United Kingdom; d Department of Vascular and Endovascular Surgery, Addenbrooke’s Hospital, Cambridge, United Kingdom; e Department of Radiology, University of Cambridge, Cambridge, United Kingdom; f Experimental Medicine and Immunotherapeutics, University of Cambridge, Cambridge, United Kingdom; g Department of Hematology, University of Cambridge, and National Health Service Blood and Transport, Cambridge Biomedical Campus, Cambridge, United Kingdom; h Division of Neurosurgery, Addenbrooke’s Hospital, Cambridge, United Kingdom; i Department of Radiology, Hammersmith Hospital, London, United Kingdom; j Department of Nuclear Medicine, Addenbrooke’s Hospital, Cambridge, United Kingdom; k Institute of Nuclear Medicine, University College London, London, United Kingdom; l Department of Human Genetics, Wellcome Trust Sanger Institute, Wellcome Trust Genome Campus, Hinxton, United Kingdom

**Keywords:** atherosclerosis, inflammation, macrophages, molecular imaging, positron emission tomography, somatostatin receptor, ACS, acute coronary syndrome, BMI, body mass index, CT, computed tomography, CVD, cardiovascular disease, ECG, electrocardiogram, FDG, fluorodeoxyglucose, ICC, intra-class coefficient, PET, positron emission tomography, SST_2_, somatostatin receptor subtype-2, TBR, tissue-to-blood ratio, TBR_max_, maximum tissue-to-blood ratios, TIA, transient ischemic attack

## Abstract

**Background:**

Inflammation drives atherosclerotic plaque rupture. Although inflammation can be measured using fluorine-18-labeled fluorodeoxyglucose positron emission tomography ([^18^F]FDG PET), [^18^F]FDG lacks cell specificity, and coronary imaging is unreliable because of myocardial spillover.

**Objectives:**

This study tested the efficacy of gallium-68-labeled DOTATATE (^68^Ga-DOTATATE), a somatostatin receptor subtype-2 (SST_2_)-binding PET tracer, for imaging atherosclerotic inflammation.

**Methods:**

We confirmed ^68^Ga-DOTATATE binding in macrophages and excised carotid plaques. ^68^Ga-DOTATATE PET imaging was compared to [^18^F]FDG PET imaging in 42 patients with atherosclerosis.

**Results:**

Target *SSTR2* gene expression occurred exclusively in “proinflammatory” M1 macrophages, specific ^68^Ga-DOTATATE ligand binding to SST_2_ receptors occurred in CD68-positive macrophage-rich carotid plaque regions, and carotid *SSTR2* mRNA was highly correlated with in vivo ^68^Ga-DOTATATE PET signals (r = 0.89; 95% confidence interval [CI]: 0.28 to 0.99; p = 0.02). ^68^Ga-DOTATATE mean of maximum tissue-to-blood ratios (mTBR_max_) correctly identified culprit versus nonculprit arteries in patients with acute coronary syndrome (median difference: 0.69; interquartile range [IQR]: 0.22 to 1.15; p = 0.008) and transient ischemic attack/stroke (median difference: 0.13; IQR: 0.07 to 0.32; p = 0.003). ^68^Ga-DOTATATE mTBR_max_ predicted high-risk coronary computed tomography features (receiver operating characteristics area under the curve [ROC AUC]: 0.86; 95% CI: 0.80 to 0.92; p < 0.0001), and correlated with Framingham risk score (r = 0.53; 95% CI: 0.32 to 0.69; p <0.0001) and [^18^F]FDG uptake (r = 0.73; 95% CI: 0.64 to 0.81; p < 0.0001). [^18^F]FDG mTBR_max_ differentiated culprit from nonculprit carotid lesions (median difference: 0.12; IQR: 0.0 to 0.23; p = 0.008) and high-risk from lower-risk coronary arteries (ROC AUC: 0.76; 95% CI: 0.62 to 0.91; p = 0.002); however, myocardial [^18^F]FDG spillover rendered coronary [^18^F]FDG scans uninterpretable in 27 patients (64%). Coronary ^68^Ga-DOTATATE PET scans were readable in all patients.

**Conclusions:**

We validated ^68^Ga-DOTATATE PET as a novel marker of atherosclerotic inflammation and confirmed that ^68^Ga-DOTATATE offers superior coronary imaging, excellent macrophage specificity, and better power to discriminate high-risk versus low-risk coronary lesions than [^18^F]FDG. (Vascular Inflammation Imaging Using Somatostatin Receptor Positron Emission Tomography [VISION]; NCT02021188)

Systemic inflammation triggers culprit pathogenic mechanisms, relating clinical cardiovascular disease (CVD) risk factors to atherosclerotic plaque progression and rupture (1). Quantifying vascular inflammation in atherosclerosis may reveal mechanistic pathways, allow efficacy testing of new drugs, and improve CVD risk prediction.

Carotid, aortic, and peripheral arterial inflammation can be measured by fluorine-18-labeled fluorodeoxyglucose positron emission tomography/computed tomography ([^18^F]FDG PET/CT) (2). However, myocardial [^18^F]FDG signal spillover occurs due to myocardial muscle [^18^F]FDG uptake, often hampering coronary artery signal quantification (3). Lack of cell specificity and the influence of hypoxia on [^18^F]FDG uptake within macrophages and other cells (4) are further limitations of [^18^F]FDG imaging.

Up-regulation of the G-protein-coupled receptor somatostatin receptor subtype-2 (SST_2_) occurs on the surface of activated macrophages (5). Pre-clinical 6, 7 and retrospective 8, 9, 10 studies suggest that gallium-68-labeled [1,4,7,10-tetraazacyclododecane-*N*,*N*',*N*'',*N*'''-tetraacetic acid]-d-Phe^1^, Tyr^3^-octreotate (DOTATATE), a PET ligand with high-specificity binding affinity for SST_2_ (11), may be superior to [^18^F]FDG in marking macrophage activity, particularly in the coronary arteries. However, robust evaluation of ^68^Ga-DOTATATE in atherosclerosis is lacking.

We present a prospective clinical study evaluating ^68^Ga-DOTATATE PET for imaging coronary, carotid, and aortic inflammation in patients with CVD.

## Methods

### RNA sequencing

To determine target specificity of ^68^Ga-DOTATATE imaging in atherosclerosis, expression of the *SSTR1–5* genes within in vitro- differentiated macrophage subtypes and other blood-derived cells relevant to atherosclerosis were characterized using population-based “next generation” RNA sequencing data from the European BLUEPRINT (a BLUEPRINT of haematopoietic epigenomes) project, for which all data are publicly available (12). The expression levels of *glucose transporter 1* (*GLUT1*) and *glucose transporter 3* (*GLUT3*) genes were also analyzed from the dataset; these genes encode the main glucose transporters that facilitate uptake of [^18^F]FDG in atherosclerotic plaques.

### Clinical study

**Figure 1:**
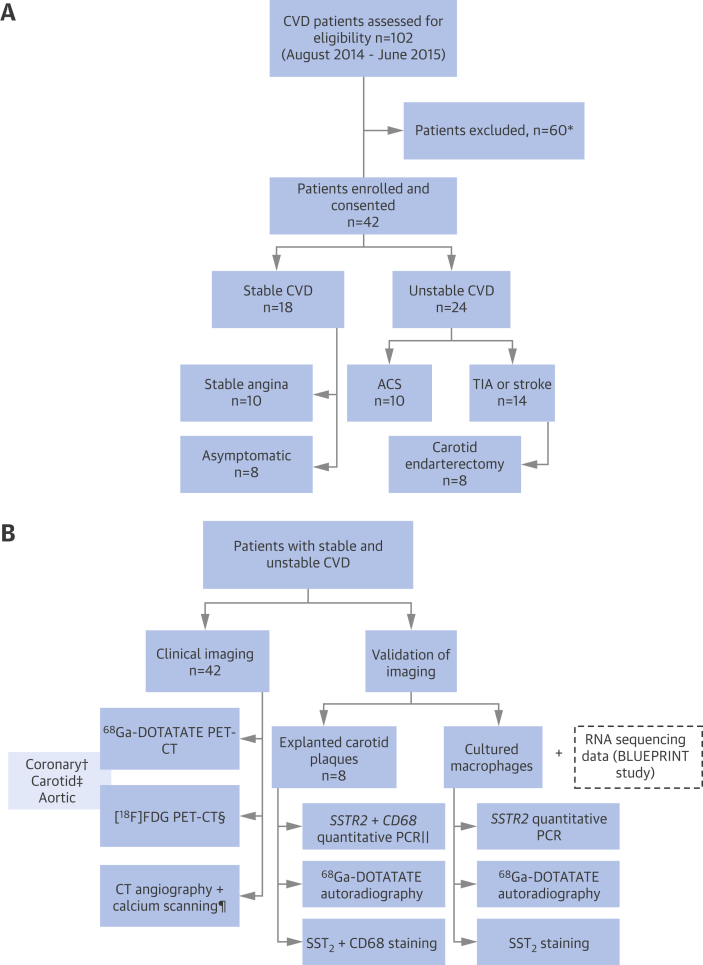
The VISION Study Patient **(A)** and procedure **(B)** flowcharts. ∗Did not meet study criteria, n = 8; other clinical factors, n = 3; declined/cancelled, n = 49. †Coronary artery PET data excluded in ACS patients with ambiguous culprit arteries (n = 2). ‡Carotid artery PET data excluded in patients with prior carotid surgery (n = 2). §[^18^F]-FDG PET imaging not completed because of timing of surgery (n = 1). ‖Tissue samples excluded owing to insufficient mRNA extracted for quantitative PCR (n = 2). ¶CT scans not completed (calcium scan, n = 1; coronary angiogram, n = 5; carotid angiogram, n = 2). ACS = acute coronary syndrome; CT = computed tomography; CVD = cardiovascular disease; FDG = fluorodeoxyglucose; PCR = polymerase chain reaction; PET = positron emission tomography; TIA = transient ischemic attack; VISION = Vascular Inflammation imaging using Somatostatin receptor positron emissION tomography.

In the VISION (Vascular Inflammation imaging using Somatostatin receptor positron emissION tomography; NCT02021188) study, an unselected “real-world” cohort of patients with wide-ranging severity of stable (n = 18) and unstable (n = 24) CVD was prospectively enrolled from Addenbrooke’s Hospital, Cambridge, United Kingdom (Figure 1). “Stable” patients had stable angina or asymptomatic atherosclerosis and at least a 30% stenosis of a major epicardial coronary artery or an internal carotid artery. “Unstable” patients had experienced a clinical event (acute coronary syndrome [ACS] or carotid territory transient ischemic attack [TIA]/stroke) within the 3 months before imaging. Baseline cardiovascular risk factors were noted, including measurement of serum lipids and high-sensitivity C-reactive protein. Patients were older than 40 years of age and provided written, informed consent. The study protocol approved by the local research ethics committee (REC 14/EE/0019) was in accordance with the Declaration of Helsinki.

### PET-CT imaging

Patients underwent ^68^Ga-DOTATATE PET-CT and [^18^F]FDG PET-CT imaging, using established methods (13) for vascular PET imaging on a Discovery 690 combined PET-CT system model (GE Healthcare, Little Chalfont, United Kingdom; extended Methods are detailed in the Online Appendix). ^68^Ga-DOTATATE had an average radiochemical purity of 99% on quality control testing performed by the manufacturer (Mallinckrodt, St. Louis, Missouri). Patients fasted for 6 h prior to [^18^F]FDG imaging; capillary blood glucose concentration was confirmed as <7.0 mmol/l in nondiabetic patients prior to scanning. Patients with diabetes mellitus were instructed to take their antidiabetic medications as usual prior to [^18^F]FDG scanning but to hold insulin within 4 h of the scan; if glucose level was >11.0 mmol/l, the scan was rescheduled according to our standard clinical practice. Insulin was not administered to any patient prior to [^18^F]FDG PET imaging. The mean injected dose of ^68^Ga-DOTATATE was 147.8 ± 31.6 MBq and 248.1 ± 22.3 MBq for [^18^F]FDG. Electrocardiography (ECG)-gated CT coronary angiography plus calcium scanning and carotid angiography were also performed.

### Image analysis

Static PET images were reconstructed using 3-dimensional (3D) iterative time-of-flight ordered-subset expectation maximization with point spread function modeling to reduce partial volume error. ECG-gated coronary PET images were reconstructed in diastole (50% to 75% of the R–R interval). PET-CT images were coregistered and analyzed by experienced observers masked to the clinical details, using OsiriX imaging software (version 7.0; Pixmeo, Bernex, Switzerland). CT angiography was used as the anatomical reference standard; 2D regions of interest were drawn on consecutive, fused PET-CT slices to quantify the maximum arterial radioactivity concentration, normalized by mean blood pool activity in the superior vena cava (maximum tissue-to-blood ratio [TBR_max_]). Mean (m) and most diseased segment (mds) TBR_max_ values were measured for each coronary segment, carotid artery, and thoracic aorta. Reproducibility of ^68^Ga-DOTATATE TBR measurements were tested by 2 independent observers using 10% of the coronary and carotid scans (n = 4 for both) selected at random, with 1 week between intraobserver readings. Coronary artery PET data were deemed uninterpretable if the maximum myocardial standardized uptake value was >5.0.

Coronary lesions were classified according to established CT criteria for plaque composition (calcified, noncalcified, or mixed plaque) and the presence of high-risk morphological features (spotty calcification [<3.0 mm], low attenuation [<30 HU], and positive remodeling [cross-sectional area >10% of a reference segment]) (14).

“Culprit” lesions were defined in patients with ACS or TIA/stroke by the attending cardiologist or stroke physician before PET imaging, with no involvement of the VISION study team. Assignment of culprit artery status took clinical data into consideration (e.g., ECG, angiographic and echocardiographic findings, site of any neurological deficit at time of clinical presentation, and carotid artery or brain imaging). Arteries targeted for intervention (with percutaneous coronary intervention or carotid endarterectomy surgery) were presumed to be culprit arteries. In patients who were managed medically, if the culprit lesion was uncertain, the relevant data were excluded from this part of the analysis.

### Quantitative polymerase chain reaction

The pattern of *SSTR2* gene expression observed using population-based RNA-sequenced data was confirmed in lipopolysaccharide-stimulated macrophages from subjects in our imaging cohort by using quantitative real-time polymerase chain reaction assay results and compared to those of age- and sex-matched healthy volunteers (n = 3 for both). *SSTR2* and *CD68* mRNA levels were measured in excised carotid plaques, and compared with ^68^Ga-DOTATATE signals in PET images obtained prior to surgery.

### Autoradiography and histology

To confirm specific ligand binding in atherosclerotic plaques, ^68^Ga-DOTATATE autoradiography was performed in carotid tissue sections adjacent to those used for quantitative polymerase chain reaction. After the radioactivity decayed, sections were stained with antibodies for SST_2,_ the panmacrophage marker CD68, and Movat’s pentachrome stain for anatomic characterization. Autoradiography and immunostaining were similarly tested in cultured macrophages. Colocalization of SST_2_ and CD68 staining in macrophages within carotid plaque sections were assessed by immunofluorescence, with isotype and concentration-matched immunoglobulin G (IgG) as the negative control. The retention, storage, and use of tissue sections and blood samples were compliant with the UK Human Tissue Act of 2004.

### Statistical analysis

The primary outcome was comparison of culprit versus nonculprit coronary and carotid artery ^68^Ga-DOTATATE mTBR_max_ in patients with ACS or TIA/stroke. Pre-specified secondary outcomes included comparisons of vascular ^68^Ga-DOTATATE mTBR_max_ values versus clinical CVD risk factors, CT plaque morphology, [^18^F]FDG mTBR_max_, and *SSTR2*/*CD68* gene expression levels in excised carotid plaques. Primary and secondary outcome data expressed as medians (interquartile range [IQR]) were compared using Wilcoxon signed rank test or Mann-Whitney *U* test, as appropriate, with differences of medians derived for paired data. For comparisons between more than 2 groups, the Kruskal-Wallis test was used.

Based on ^68^Ga-DOTATATE TBR values from our pilot work and previously published data (9), our sample size (n = 42) was chosen to detect differences in mTBR_max_ of ≥1.13 between high- and low-risk arteries, with 80% power and a 2-sided p value of <0.05. Patients with stable and unstable CVD were not formally matched as our primary comparison used “within patient” data (culprit versus nonculprit artery) rather than stable versus unstable patients. We anticipated that if one-third of patients had TIA/stroke, this would yield a comparable number of explanted carotid specimens to similar PET validation work performed by our group (15).

Spearman’s correlation and simple linear regression were used to identify statistically significant clinical and biochemical predictors of ^68^Ga-DOTATATE mTBR_max_ that were then evaluated together using multivariate analysis. In the regression analysis, mean arterial values were used to mitigate the problem of multiple observations, as each patient contributed an equal number of arteries. Two-sided p values of <0.05 were considered significant. Statistical analysis was performed using Prism version 6.0 software (GraphPad Software, Redwood, California) and Stata version 14.1 software (StataCorp, Cary, North Carolina).

## Results

### Population-based validation of *SSTR2* gene expression in macrophages

**Figure 2:**
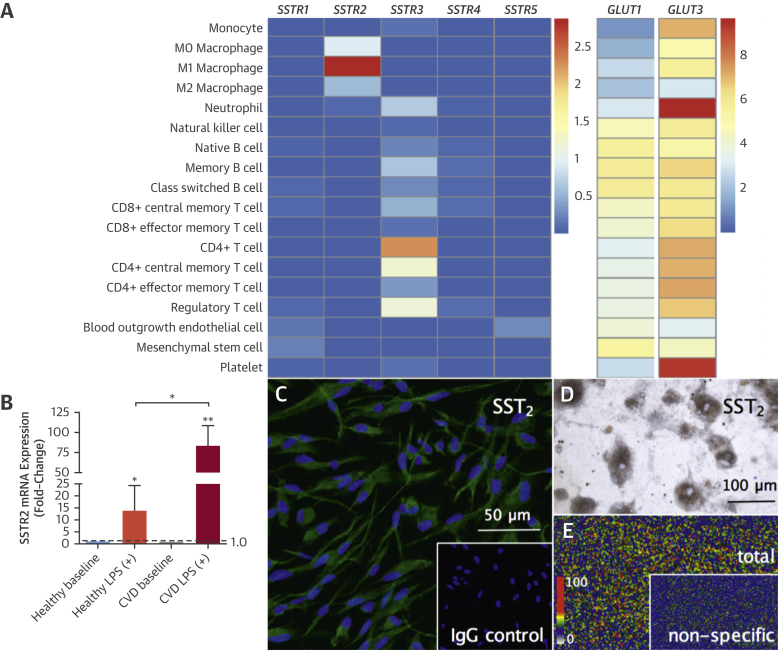
Target *SSRT2* Expression in Proinflammatory Macrophages Heatmap of population-based RNA sequencing data **(A)** showing high *SSTR2* expression in proinflammatory M1 macrophages (n = 4), very low levels of *SSTR2* expression in unstimulated M0 macrophages (n = 4), and alternatively activated M2 macrophages (n = 5). For comparison, a heatmap of *GLUT1* and *GLUT3* shows significant gene expression in all cell types (note, different scales for *SSRT* and *GLUT* genes; mean values are log2 fragments per kilobase of transcript for million mapped reads [FPKM+1]). *SSTR2* expression in LPS-stimulated macrophages from CVD patients versus age- and sex-matched healthy volunteers (n = 3 for both) using quantitative PCR **(B)**. Photomicrograph shows **green** fluorescent immunoreactive SST_2_ staining in macrophages **(C)**, with **blue** nuclear DAPI-stained ([**inset**] concentration and isotype-matched IgG negative control). Brightfield photomicrograph shows **brown** immunoreactive SST_2_-stained cultured macrophages, with nuclear counterstain **(D)**. Phosphor autoradiographic image shows total binding of ^68^Ga-DOTATATE **(E)** in clusters of cultured macrophages ([**inset**] parallel incubation with ^68^Ga-DOTATATE and cold competing ligand showing very low levels of nonspecific binding). IgG = immunoglobulin G; LPS = lipopolysaccharide; other abbreviations as in Figure 1.

Prior to clinical PET imaging, we tested the target expression of *SSTR2* in blood-derived macrophages compared to other relevant cell types by using data from a large-scale population study. High levels of *SSTR2* mRNA were detected exclusively in proinflammatory M1 macrophages and no other macrophage phenotype. This pattern and degree of expression was not seen for any other SST receptor subtype or cell line (Figure 2). *SSTR3* was expressed by CD4^+^ T lymphocytes to a lesser extent, as is known to occur (16).

Very low levels of *SSTR2* mRNA were detected in unstimulated M0 macrophages and alternatively activated M2 macrophages, but *SSTR2* was not expressed by any of the following cells: monocytes, T or B lymphocytes, natural killer cells, platelets, neutrophils, and endothelial cells. *GLUT1* and *GLUT3* were highly expressed by all cell types, demonstrating that *SSTR2* offers greater cell specificity as an inflammation imaging target than glucose metabolism.

### Clinical study

Baseline clinical data are summarized in Table 1. The median time interval between ACS and PET imaging was 35 days (IQR: 21 to 66 days) and 18 days (IQR: 11 to 25 days) for TIA/stroke.

The reproducibility of ^68^Ga-DOTATATE TBR_max_ measurements was excellent for both intraobserver observations (coronary artery intraclass coefficient value [ICC]: 0.90; 95% confidence interval [CI]: 0.85 to 0.94; carotid artery ICC: 0.96; 95% CI: 0.95 to 0.97) and interobserver observations (coronary artery ICC: 0.96; 95% CI: 0.94 to 0.97; carotid artery ICC: 0.91; 95% CI: 0.88 to 0.94).

### ^68^Ga-DOTATATE identifies culprit ACS lesions in coronary arteries

Myocardial binding of ^68^Ga-DOTATATE was sufficiently low in all patients to allow unimpeded coronary artery PET signal measurement (Central Illustration, Online Figure 1). In patients with ACS, culprit ^68^Ga-DOTATATE uptake was consistently greater than the highest nonculprit coronary segment within the same individual (median difference mTBR_max_: 0.69; IQR: 0.22 to 1.15; p = 0.008; median difference mdsTBR_max_: 1.17; IQR: 0.45 to 1.70; p = 0.02), regardless of whether the lesion had been stented prior to imaging (culprit stented mTBR_max_: 2.91; IQR: 2.66 to 4.63 vs. stable stented mTBR_max_: 2.00; IQR: 1.51 to 2.70; p = 0.006) (Online Figure 2).

Using receiver operator characteristic (ROC) analysis, coronary ^68^Ga-DOTATATE mTBR_max_ values >2.66 had 87.5% (95% CI: 47.4 to 99.7) sensitivity and 78.4% (95% CI: 72.4 to 83.6) specificity to detect a culprit coronary segment (ROC area under the curve [AUC]: 0.86; 95% CI: 0.78 to 0.93; p = 0.0006).

### ^68^Ga-DOTATATE identifies high-risk stable lesions in coronary arteries

**Figure 3:**
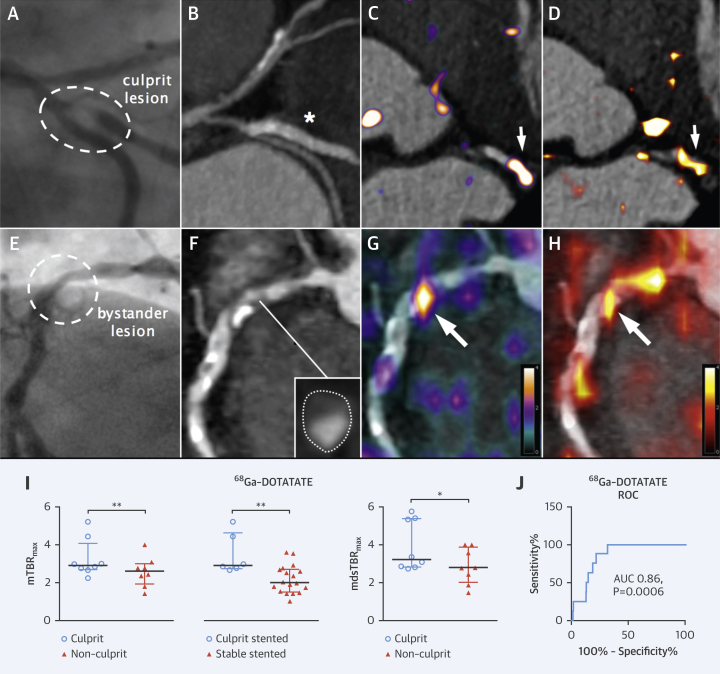
Coronary PET Inflammation Imaging: ACS Culprit Versus Bystander Lesions X-ray angiography images from a 59-year old man with ACS, showing a culprit first obtuse marginal lesion (**[A] hatched oval**) and nonculprit (bystander) right coronary artery disease ([**E**] **circle**). Identification of a culprit artery was aided by electrocardiographic findings of lateral T-wave inversion. Corresponding CT angiography images **(B, F)** show stented culprit lesion (*) and native bystander lesion with high-risk plaque morphology ([**inset**] low attenuation, cross-section of artery with outer wall boundary marked by **dotted outline**). In both lesions, intense inflammation **(arrows)** detected by ^68^Ga-DOTATATE PET **(C, G)** is reproduced by [^18^F]FDG PET **(D, H)**. Graphs of culprit versus highest nonculprit coronary ^68^Ga-DOTATATE TBR_max_ values in patients (n = 8) with ACS and stented culprit ACS lesions (n = 6) versus stable stented (n = 18) lesions **(I)**. ROC analysis demonstrates good diagnostic accuracy of ^68^Ga-DOTATATE for culprit coronary lesions **(J)**. Note stable stented lesions are coronary stents that were inserted >3 months prior to PET imaging in all but 1 patient. AUC = area under curve; mTBR_max_ = mean of maximum tissue-to-blood ratios; ROC = receiver operating characteristic; other abbreviations as in Figures 1 and 2.

**Figure 4:**
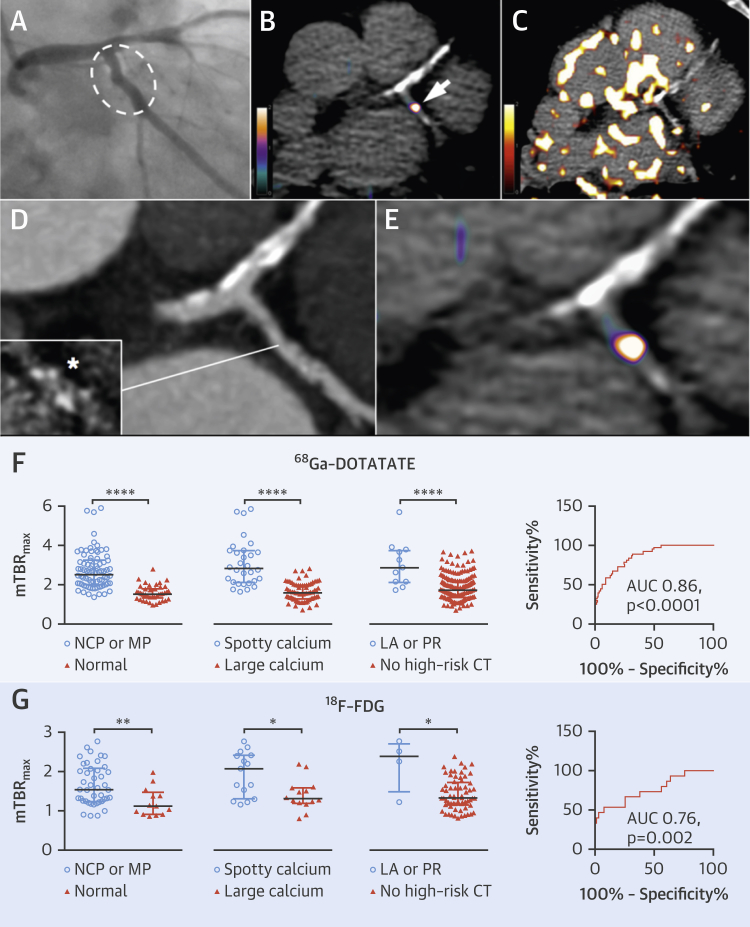
Coronary PET Inflammation Imaging: High-Risk CT Features **(A)** X-ray and (**D**) CT coronary angiograms of a 67-year-old man with stable angina, showing minor LCx atheroma (**hatched oval**) with spotty calcification ([**inset**] *calcium scan) and calcified plaque in the LAD artery. Although ^68^Ga-DOTATATE PET **(B, E)** allows unimpeded interpretation of inflammation in the LCx lesion (**B**, **arrow**), and lack of signal in the LAD, coronary [^18^F]FDG imaging is obscured by patchy myocardial tracer uptake (**C**). Graphs compare ^68^Ga-DOTATATE **(F)** with [^18^F]FDG (**G**) coronary TBR_max_ values by CT plaque morphology in coronary segments (^68^Ga-DOTATATE: NCP or MP, n = 86; normal, n = 45; spotty calcium, n = 30; large calcium, n = 72; LA or PR, n = 11; no high-risk CT, n = 186; [^18^F]FDG: NCP or MP, n = 43; normal, n = 13; spotty calcium, n = 15; large calcium n = 14; LA or PR, n = 4; no high-risk CT, n = 66), and ROC analysis demonstrating good diagnostic accuracy for high-risk coronary lesions. LA = low attenuation; LAD = left anterior descending; LCx = left circumflex; NCP = noncalcified plaque; MP = mixed plaque; PR = positive remodeling; other abbreviations in Figures 1, 2, and 3.

Data from 6 ± 2 coronary segments were analyzed from each patient. Increased ^68^Ga-DOTATATE signals were often observed in nonculprit (bystander) lesions in ACS patients, particularly in low-attenuation plaques defined by CT (Figure 3). ^68^Ga-DOTATATE mTBR_max_ values were higher in nonculprit coronary segments in patients with both stable and unstable CVD, with either noncalcified/mixed plaque morphology or with high-risk CT features (spotty calcification, low attenuation, or positive remodeling) versus heavily calcified or normal arteries with no high-risk features (p < 0.0001) (Figure 4).

Coronary ^68^Ga-DOTATATE mTBR_max_ >2.12 had 83.3% (95% CI: 67.2% to 93.6%) sensitivity and 71.7% (95% CI: 64.6% to 78.0%) specificity (ROC AUC: 0.86; 95% CI: 0.80 to 0.92; p < 0.0001) to detect a segment with at least 1 high-risk CT feature.

### ^68^Ga-DOTATATE identifies culprit TIA/stroke lesions in carotid arteries

**Figure 5:**
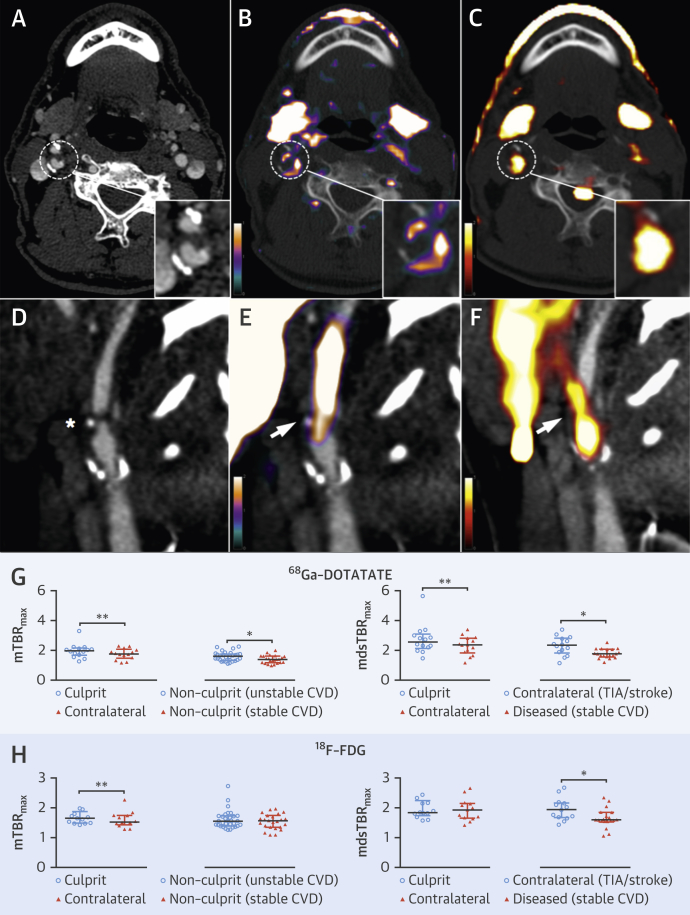
Carotid PET Inflammation Imaging: TIA/Stroke Views from a 66-year-old man ([**top**] axial plane) and a 70-year-old man ([**bottom**] sagittal plane), both of whom had TIAs resulting from right internal carotid artery lesions, shown on CT **(A [hatched circle], D [∗])**, with intense culprit plaque inflammation (**hatched circles/arrows**) detected by ^68^Ga-DOTATATE **(B, E)** and reproduced by [^18^F]FDG (**C, F**). Graphs compare culprit versus nonculprit ^68^Ga-DOTATATE **(G)** and [^18^F]FDG (**H**) TBR_max_ values (^68^Ga-DOTATATE: culprit n = 14; contralateral n = 14; nonculprit [unstable CVD] n = 31; nonculprit [stable CVD] n = 24; contralateral [TIA/stroke] n = 14; diseased stable CVD n = 19; [^18^F]FDG: culprit n = 13; contralateral n = 13; nonculprit [unstable CVD] n = 31; nonculprit [stable CVD] n = 24; contralateral [TIA/stroke] n = 13; diseased [stable CVD] n = 19). TIA = transient ischemic attack; other abbreviations as in Figures 1, 2, and 3.

In patients with TIA or stroke, increased ^68^Ga-DOTATATE inflammatory signals reliably differentiated between culprit carotid plaques and contralateral nonculprit carotid arteries (median difference mTBR_max_: 0.13; IQR: 0.07 to 0.32; p = 0.003; median difference mdsTBR_max_: 0.34; IQR: −0.01 to 0.53; p = 0.005) (Figure 5). Contralateral carotid ^68^Ga-DOTATATE mdsTBR_max_ in patients with TIA/stroke was also greater than in diseased (p = 0.01) or normal (p = 0.0001) carotids from patients with stable CVD (i.e., those without TIA/stroke or ACS). Nonculprit carotid ^68^Ga-DOTATATE mTBR_max_ was also higher in patients with unstable CVD (either TIA/stroke or ACS) versus stable CVD (p = 0.02).

### Aortic ^68^Ga-DOTATATE signals are related to coronary ^68^Ga-DOTATATE signals

^68^Ga-DOTATATE mTBR_max_ values in the coronary arteries and neighboring aorta showed a moderate correlation (r = 0.43; 95% CI: 0.11 to 0.66; p = 0.008). Aortic ^68^Ga-DOTATATE mTBR_max_ was negatively correlated with coronary calcium scores in patients with a total score of <400 (r = −0.66; 95% CI: −0.87 to 0.26; p = 0.003).

### Vascular ^68^Ga-DOTATATE signals are related to clinical CVD risk factors

**Figure 6:**
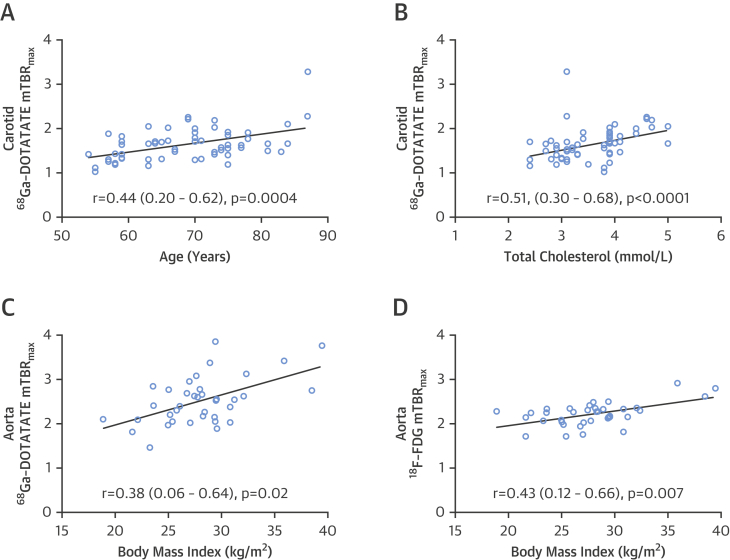
Vascular Inflammation Versus Clinical Risk Factors Graphs show correlations of vascular inflammation detected by ^68^Ga-DOTATATE **(A to C)** and [^18^F]FDG **(D)** versus clinical cardiovascular disease risk factors. (Carotid arteries n = 62; aortas, n = 38; note data from patients not taking statins [n = 4] were excluded to control for this variable). Abbreviations as in Figure 1.

Relationships between vascular ^68^Ga-DOTATATE signals and clinical CVD risk factors were evaluated to explore possible mechanistic links between ^68^Ga-DOTATATE and underlying disease pathology. Age (r = 0.44; 95% CI: 0.20 to 0.62; p = 0.0004), total cholesterol (r = 0.51; 95% CI: 0.30 to 0.68]; p < 0.0001), and Framingham risk score (r = 0.53; 95% CI: 0.32 to 0.69; p <0.0001) showed significant correlations with carotid ^68^Ga-DOTATATE mTBR_max_ (Figure 6). Carotid ^68^Ga-DOTATATE mTBR_max_ also differed significantly across patients grouped according to Framingham risk score (p < 0.0001). Body mass index (BMI) was positively correlated with aortic ^68^Ga-DOTATATE mTBR_max_ (r = 0.38; 95% CI: 0.06 to 0.64; p = 0.017). When age, total cholesterol, and BMI were evaluated with other relevant clinical factors using multivariate linear regression, they remained significant predictors of ^68^Ga-DOTATATE mTBR_max_ (Online Table 1).

**Figure 7:**
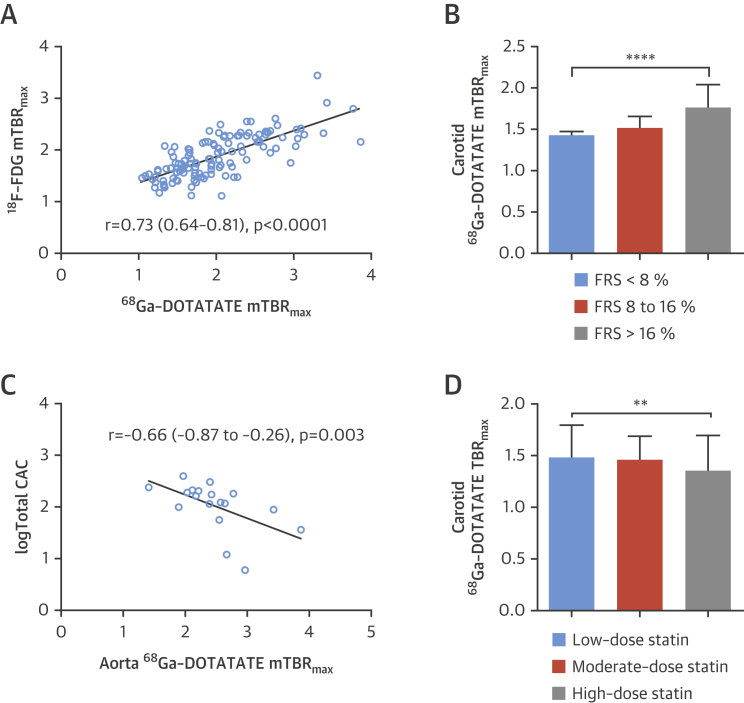
^68^Ga-DOTATATE Versus [^18^F]FDG-Defined Inflammation and Other Clinical Factors Graphs show **(A)** the strong correlations among coronary, carotid, and aortic ^68^Ga-DOTATATE mTBR_max_ versus [^18^F]FDG mTBR_max_ (n = 123 mean arterial values per tracer); **(B)** carotid ^68^Ga-DOTATATE mTBR_max_ grouped by FRS (<8%, n = 16; 8% to 16%, n = 14; >16%, n = 32); **(C)** negative correlation of coronary aortic mTBR_max_ versus calcium score in patients with CAC <400 (n = 19); and **(D)** carotid ^68^Ga-DOTATATE TBR_max_ ROI values in non TIA/stroke patients grouped by statin dosages (n = 20 patients [14 ROIs per artery]; low-dose n = 4; moderate dose, n = 9; high-dose, n = 7). Log transformed CAC values used to account for the non-normal distribution in the general population. Median difference between low-dose vs. high-dose: −0.13; (95% CI: −0.17 to −0.016); p = 0.02; %Δ −8.65. Low dose: simvastatin, 20 mg or lower; high-dose: atorvastatin, 80 mg or equivalent; moderate dose: all other dosages. CAC = coronary artery calcium score; FRS = %10-year Framingham risk score; ROI = region of interest; TIA = transient ischemic attack.

Carotid ^68^Ga-DOTATATE TBR_max_ values also varied significantly in patients without TIA/stroke who were taking statins, with lower values seen in patients taking high-intensity statins compared to those taking moderate or low dosages (p = 0.004) (Figure 7).

In the 1.6 ± 0.2 years following PET imaging, 2 patients attended the emergency department with nonanginal chest pain, and there were 2 out-of-hospital deaths; our study was not powered to assess the ability of PET imaging to predict clinical events.

### Comparison of ^68^Ga-DOTATATE versus [^18^F]FDG-defined inflammation

The time interval between ^68^Ga-DOTATATE and [^18^F]FDG imaging was a median of 2 days (IQR: 1 to 7 days). Coronary, carotid, and aortic ^68^Ga-DOTATATE and [^18^F]FDG mTBR_max_ values were strongly correlated with each other (r = 0.73; 95% CI: 0.64 to 0.81; p < 0.0001), although coronary artery [^18^F]FDG data were uninterpretable in 27 (64%) patients because of high myocardial spillover. Of 2 ACS patients with interpretable coronary [^18^F]FDG data, culprit mTBR_max_ values were numerically higher than the highest nonculprit segment in 1 patient.

[^18^F]FDG mTBR_max_ but not mdsTBR_max_ differentiated culprit from contralateral carotids (median difference: 0.12; IQR: 0.00 to 0.23; p = 0.008). Comparisons between coronary [^18^F]FDG mTBR_max_ values and CT morphology are shown in Figure 4 and clinical risk factors in Figure 6. Coronary ^68^F-FDG mTBR_max_ of >2.05 had 53.3% (95% CI: 26.6% to 78.7%) sensitivity and 92.4% (95% CI: 83.2% to 97.5%) specificity (ROC AUC: 0.76; 95% CI: 0.62 to 0.91; p = 0.002) for high-risk CT features. ^68^Ga-DOTATATE demonstrated higher TBR values and superior ability to discriminate high-risk versus low-risk coronary atherosclerotic lesions than [^18^F]FDG.

### Target validation in macrophages from CVD patients

In CVD patients, macrophage *SSTR2* mRNA was increased a median 91-fold (IQR: 56 to 104) above baseline versus 13-fold (IQR: 4.0 to 25) in age- and sex-matched healthy volunteers (p = 0.01), after stimulation with lipopolysaccharide. Presence of SST_2_ receptors was confirmed by immunostaining and specific binding of ^68^Ga-DOTATATE to SST_2_ in cultured macrophages shown by autoradiography.

### Autoradiographic and histological target validation in carotid plaques

**Figure 8:**
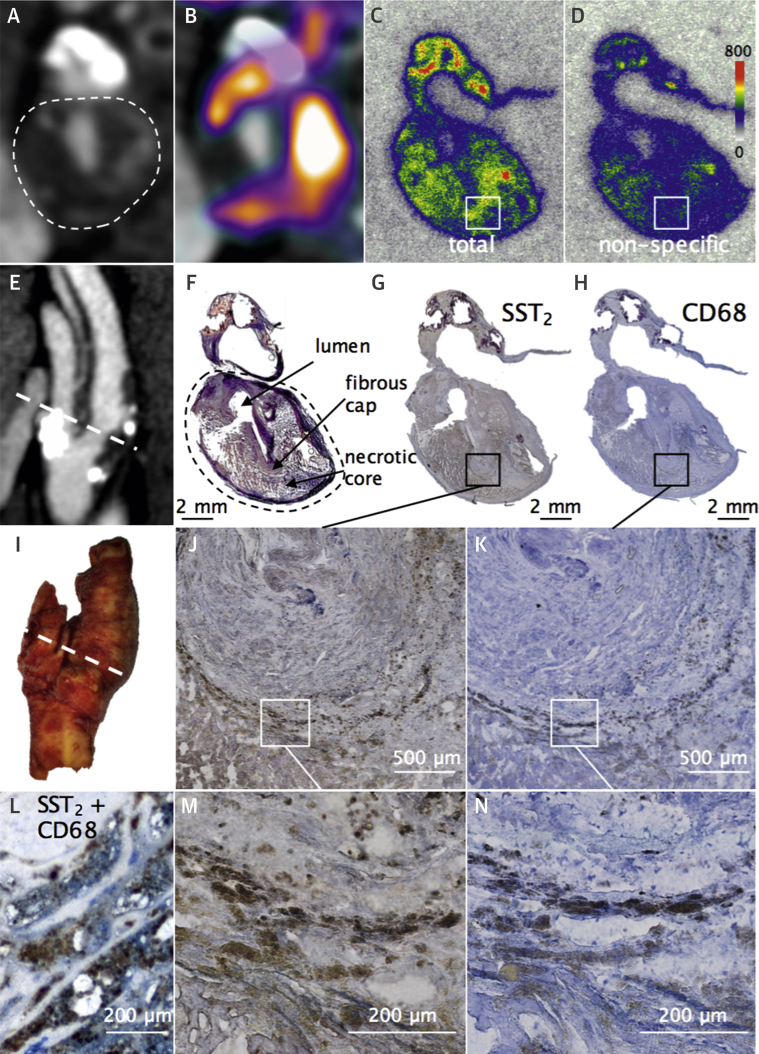
^68^Ga-DOTATATE Ligand Binding to Macrophage SST_2_ in Carotid Plaque In vivo CT angiography views of culprit carotid artery (**hatched oval** = internal carotid artery) in axial **(A)** and sagittal **(E)** views, with corresponding fused ^68^Ga-DOTATATE PET-CT **(B)**. Ex vivo views of macrographic images of the explanted carotid specimen (**I**, **hatched line** signifies location of carotid section); phosphor autoradiographic image shows the total binding of ^68^Ga-DOTATATE to SST_2_ receptors in macrophages within a transverse carotid section **(C)** corresponding to the level shown in clinical images. Adjacent section was incubated with ^68^Ga-DOTATATE and cold competing ligand **(D)** showing very low levels of nonspecific binding. Brightfield photomicrographs show brown immunoreactive SST_2_ staining **(G, J, M)** of macrophages identified with the panmacrophage marker CD68 **(H, K, N)**, colocalized SST_2_**(brown)**, and CD68 **(blue)** staining in the same section **(L)**; Movat’s pentachrome stain **(F).**

Following PET-CT imaging, 8 patients underwent carotid endarterectomy. The PET scan-to-surgery time interval was a median of 9 (IQR: 3 to 35) days. Ex vivo ^68^Ga-DOTATATE carotid autoradiography showed high levels of specific ligand binding to SST_2_ receptors in all specimens (n = 8). A small degree of nonspecific binding was seen in relation to freshly cut calcium and as a result of edge artifact, which occurs when tissue edges curl causing the ligand to remain trapped during the experiment. ^68^Ga-DOTATATE binding within carotid plaques occurred mainly in the necrotic cores and shoulder regions, where there was strong colocalization of CD68 and SST_2_ staining (Figure 8, Online Figure 3). Neither ^68^Ga-DOTATATE binding nor SST_2_ staining was observed within thick fibrous cap regions consisting mainly of vascular smooth muscle cells.

### Carotid *SSTR2*/*CD68* mRNA versus ^68^Ga-DOTATATE activity

**Figure 9:**
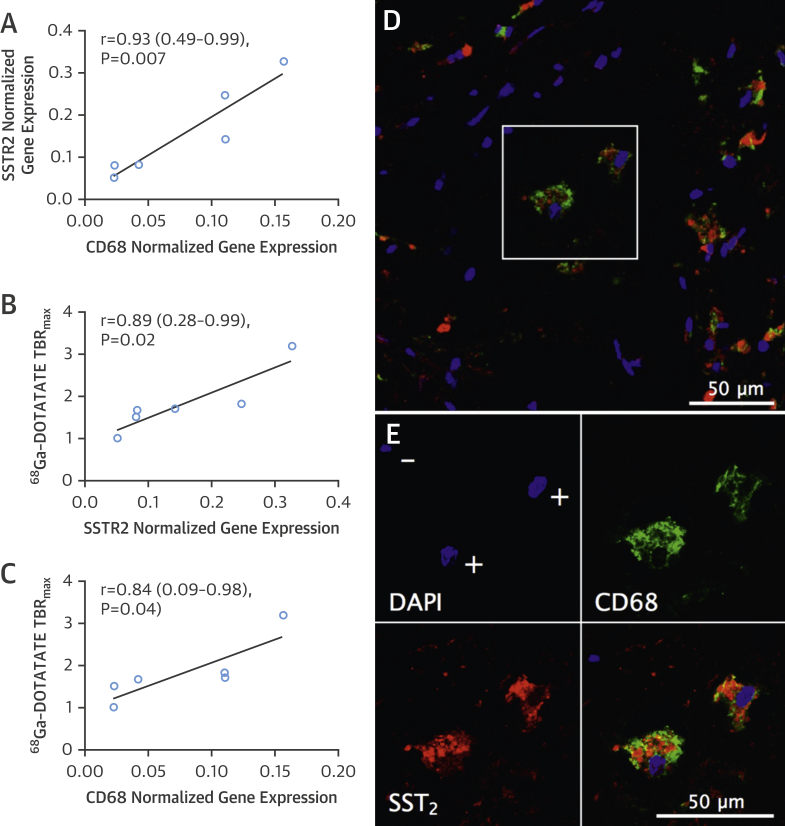
Carotid *SSTR2/CD68* mRNA Versus In Vivo ^68^Ga-DOTATATE Activity Graphs show correlations of *SSTR2* versus *CD68* mRNA within ex vivo carotid plaques measured by quantitative PCR **(A)**; *SSTR2***(B)** and *CD68***(C)** mRNA versus corresponding in vivo ^68^Ga-DOTATATE TBR_max_ values measured from clinical images (n = 6). Representative photomicrograph shows **red** SST_2_ and **green** CD68 fluorescent immunoreactive staining of macrophages within carotid plaque **(D)**, with **blue** nuclear DAPI staining. Note presence of both double positive (+) and double negative (−) staining indicating high cell specificity **(E)**.

*SSTR2* and *CD68* mRNA levels were highly correlated within carotid plaque (r = 0.93; 95% CI: 0.49 to 0.99; p = 0.007) (Figure 9). Carotid *SSTR2* and *CD68* mRNA levels also showed strong correlation with in vivo ^68^Ga-DOTATATE TBR_max_ values measured at the corresponding level in clinical PET images, orientated at the bifurcation (*SSTR2* r = 0.89; 95% CI: 0.28 to 0.99; p = 0.02; *CD68* r = 0.84; 95% CI: 0.09 to 0.98; p = 0.04). Moreover, immunofluorescence demonstrated high cell specificity of colocalized of SST_2_ and CD68 staining in carotid plaque macrophages. These data provided both histological and molecular validation of ^68^Ga-DOTATATE as a specific marker of atherosclerotic inflammation.

## Discussion

There have been previous reports of ^68^Ga-DOTATATE imaging in atherosclerosis, but they have been pre-clinical or retrospective studies, with the exception of 1 report limited to the carotid arteries. We provide the first definitive prospective validation of ^68^Ga-DOTATATE imaging as a marker of atherosclerotic inflammation.

### Which cells express *SSTR2* in atherosclerosis?

We confirmed that high target *SSTR2* gene expression occurs exclusively among activated proinflammatory M1 macrophages in atherosclerosis and demonstrated the presence of SST_2_ receptors in macrophages from patients with CVD. As a glucose analog, [^18^F]FDG lacks cell specificity, but there is some evidence that [^18^F]FDG accumulates more in M1 macrophages than in other macrophage subtypes because of differing glycolytic activity between these cells (17).

### Binding of ^68^Ga-DOTATATE within atherosclerotic plaques

We observed specific ^68^Ga-DOTATATE ligand binding to SST_2_ receptors within CD68^+^ macrophage-rich carotid plaque regions and strong correlations between carotid *SSTR2* mRNA and in vivo ^68^Ga-DOTATATE activity. Although low levels of SST_2_ expression have been previously reported in vascular smooth muscle cells, we did not observe ^68^Ga-DOTATATE binding nor SST_2_ staining within the thick fibrous cap regions where these cells are abundant, suggesting that the synthetic atherosclerotic vascular smooth muscle cell phenotype is unlikely to express SST_2_ to a degree that would be detectable by clinical imaging. These laboratory-based findings provide robust histological and molecular validation of ^68^Ga-DOTATATE as a specific marker of atherosclerotic inflammation.

### Culprit and high-risk plaque inflammation

In clinical imaging, we found that ^68^Ga-DOTATATE PET correctly identified culprit coronary and carotid arteries in individuals with ACS or TIA/stroke. The median difference between culprit and nonculprit carotid arteries was less pronounced than in coronary arteries, but these 2 regions are not necessarily directly comparable because of the high prevalence of asymptomatic contralateral carotid disease, differing imaging time points affecting tracer kinetics, and local factors determining tracer delivery and clearance. ^68^Ga-DOTATATE demonstrated reliable diagnostic accuracy to detect stable yet inflamed coronary lesions with high-risk CT morphological features.

### Systemic inflammation

The ability of ^68^Ga-DOTATATE to detect generalized vascular inflammation was shown by the close relationship between PET signals in neighboring coronary and aortic vasculature and increased inflammatory signals arising from nonculprit carotids in patients with unstable CVD. Both of these features have been previously demonstrated using [^18^F]FDG (2). Moreover, significant correlations were observed between clinical CVD risk factors and generalized vascular ^68^Ga-DOTATATE inflammatory signals, which were overall lower in patients receiving high-intensity statins and with increasing coronary calcium scores up to 400. The inverse relationship between statin dosages and signal intensity provide anecdotal evidence that ^68^Ga-DOTATATE PET may provide a useful imaging platform for monitoring the anti-inflammatory effects of atherosclerosis drugs.

### ^68^Ga-DOTATATE outperforms [^18^F]FDG

Although ^68^Ga-DOTATATE signals were strongly correlated with [^18^F]FDG-defined inflammation in multiple vascular territories, disparity between these 2 tracers reflects the fact that ^68^Ga-DOTATATE is a specific macrophage marker in atherosclerosis, whereas [^18^F]FDG provides a nonspecific measurement of glucose metabolism within plaque cells. Superiority of ^68^Ga-DOTATATE compared with [^18^F]FDG was shown by better power to discriminate high-risk versus low-risk coronary atherosclerotic lesions, higher signal-to-blood ratios, and consistently lower myocardial activity, affording clear coronary signal interpretation.

[^18^F]FDG imaging is notoriously unreliable in coronaries; in contrast, myocardial ^68^Ga-DOTATATE binding was sufficiently low to allow coronary artery inflammation imaging in all patients. ^68^Ga-DOTATATE inflammatory signals differentiated culprit from contralateral carotids, using both “mean of the whole artery” and “most-diseased segment” methods, but [^18^F]FDG only detected a difference in mean carotid uptake, hinting that ^68^Ga-DOTATATE may offer a more focal approach. ^68^Ga-DOTATATE signals also appeared more discretely localized than [^18^F]FDG signals in clinical images (Figures 3 and 5). Given the higher cost of ^68^Ga-DOTATATE than [^18^F]FDG, its use for noncoronary vascular imaging may not be justified, although in the context of research, increased macrophage specificity of ^68^Ga-DOTATATE potentially holds significant advantage for detection of subtle changes in vascular biology that may not be as clearly appreciated using a blunter imaging tool such as [^18^F]FDG.

A small number of previous studies have investigated SST_2_ PET imaging in CVD. Two studies demonstrated autoradiographic ^68^Ga-DOTATATE binding within macrophage-rich aortic atherosclerotic plaques in mice 6, 7. Five retrospective analyses of PET scans from patients who underwent imaging for oncological indications reported significant statistical relationships between vascular SST_2_ signals and clinical CVD factors, including older age, male sex, hypercholesterolemia, presence of calcified coronary plaque, prior CVD events, and Framingham risk score calculated using BMI 8, 9, 10, 18, 19. In 1 study, a strong correlation was observed between ^68^Ga-DOTATATE and [^18^F]FDG vascular TBR values, although signals from the 2 tracers did not colocalize at the sites of highest tracer uptake (9). In another study of 11 patients who underwent 3 serial ^68^Ga-DOTATATE scans following peptide receptor radionuclide therapy with lutetium-177-labeled DOTATATE, good interscan reproducibility of ^68^Ga-DOTATATE TBR measurements prior to radionuclide therapy was observed, as well as significant signal reduction 1 month after, which was most pronounced in relation to noncalcified plaques (10). These studies, although retrospective and without CT angiography or ECG-gating, are consistent with our findings.

Our finding that SST_2_ PET can differentiate culprit from contralateral carotid arteries is supported by another study of ^64^Cu-DOTATATE PET cardiac magnetic resonance in 10 patients with carotid TIA/stroke (20). However, in that study, correlation between carotid copper-64-labeled DOTATATE signals and gene expression of the monocyte/macrophage marker CD163 was observed using a multivariate regression model, leading the authors to conclude that this tracer reports on alternatively activated M2 macrophages. As hemoglobin-haptoglobin scavenging by CD163 in the setting of intraplaque hemorrhage directs monocyte differentiation toward an atheroprotective M2 phenotype (21), the finding of increased *CD163* mRNA within advanced ruptured plaques is unsurprising. However, there is no current evidence to indicate that significant *SSTR2* expression occurs in M2 macrophages. Our findings agree with those of previous work indicating that ^68^Ga-DOTATATE signals in atherosclerosis occur because of intracellular tracer accumulation following cell surface binding and receptor internalization among dense clusters of classically activated M1 macrophages (22).

Next steps involve testing in a larger, longitudinal study with clinical outcomes, similar to the ongoing BioImage (NCT00738725) and PESA (Progression of Early Subclinical Atherosclerosis; NCT01410318) [^18^F]FDG studies.

### Study limitations

Limitations of our study include inherent technical challenges of vascular PET imaging, namely low spatial resolution (∼5 mm) and image artifacts created by cardiorespiratory motion that are confounded by the high positron energy of ^68^Ga (E_max_: 1.9 MeV; average positron range: 2.4 mm). To overcome these difficulties, we used CT angiography for precise anatomical PET signal localization (spatial resolution: 0.5 to 0.6 mm), ECG-gated PET reconstruction to reduce the impact of motion, and iterative time-of-flight reconstruction with point spread function modeling to provide resolution recovery and reduce partial volume error. Coronary signal-to-noise ratio could potentially be improved further by motion correction methods in active development (23).

We did not attempt myocardial suppression of [^18^F]FDG using dietary manipulation or prolonged fasting, because in our experience, these methods are ineffective in ∼50% of cases (3) and are inconvenient for patients. Nevertheless, others have reported success using these methods.

Most of the ACS patients underwent stenting prior to PET imaging and persistence of procedure-related inflammation could have, in theory, augmented inflammatory signals detected in culprit coronary lesions. Clinical identification of culprit arteries can be challenging, particularly in the coronary arteries; although intravascular imaging can be used to confirm plaque rupture, this investigation was not performed in any of the patients in this study. Last, although the novel finding of increased *SSTR2* expression in LPS-stimulated macrophages from patients with CVD versus healthy volunteers is intriguing, further testing in a larger patient cohort is needed.

## Conclusions

We provide gene-, cell-, plaque-, and patient-level data demonstrating that SST_2_ PET imaging using ^68^Ga-DOTATATE provides a quantifiable, cell-specific marker of atherosclerotic inflammation that outperforms [^18^F]FDG in the coronary arteries. Further work is needed to confirm these findings in a larger patient population and to compare imaging with clinical outcomes. ^68^Ga-DOTATATE PET offers measurement of both generalized atherosclerotic disease activity and detailed information about local plaque functional phenotype to complement multimodal assessments of anatomic, morphologic, and hemodynamic disease severity. This approach, in selected patient populations, has the potential to improve CVD risk prediction, allowing personalized tailoring of therapies aimed to improve clinical outcomes.

Perspectives **COMPETENCY IN MEDICAL KNOWLEDGE:** ^68^Ga-DOTATATE binds to somatostatin receptor-2 (SST2) in activated inflammatory macrophages, and the tissue-to-blood ratios of ^68^Ga-DOTATATE distinguishes culprit from nonculprit coronary and carotid arteries in patients with ACS, stroke, or TIA. Although [^18^F]FDG also differentiates these types of arterial lesions, myocardial spillover renders coronary [^18^F]FDG PET scans uninterpretable in a high proportion of patients.

**TRANSLATIONAL OUTLOOK:** Future research should explore the utility of ^68^Ga-DOTATATE PET imaging of inflammation to classify patients for more aggressive therapeutic intervention and explore potential application to other inflammatory cardiovascular diseases.

## Figures and Tables

**Central Illustration:**
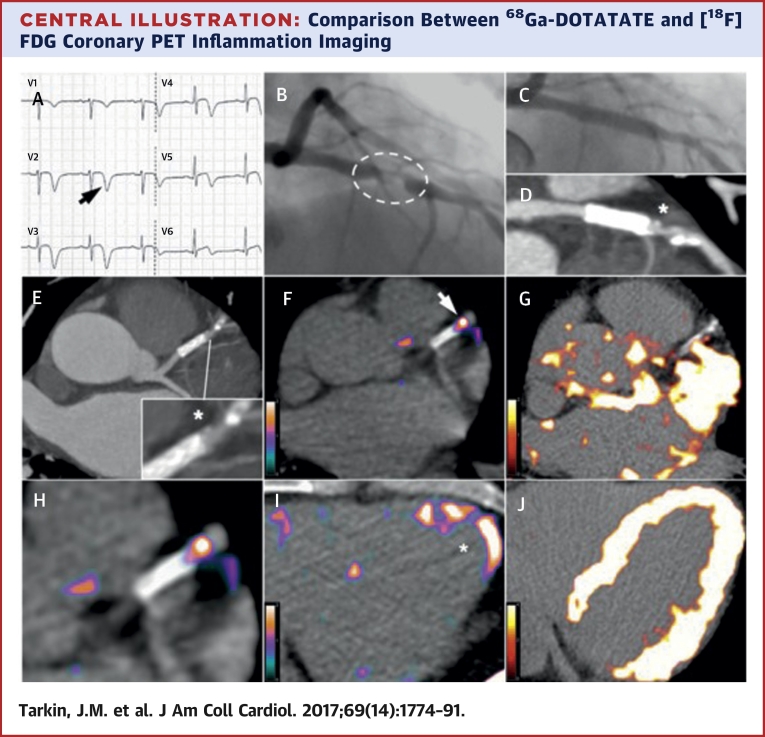
Comparison Between ^68^Ga-DOTATATE and [^18^F]FDG Coronary PET Inflammation Imaging Images from a 57-year old man with acute coronary syndrome who presented with deep anterolateral T-wave inversion **(arrow)** on electrocardiogram **(A)** and serum troponin-I concentration elevated at 4,650 ng/l (NR: <17 ng/l). Culprit left anterior descending artery stenosis **(dashed oval)** was identified by X-ray angiography **(B)**. After the patient underwent percutaneous coronary stenting **(C)**, residual coronary plaque (*inset) with high-risk morphology (low attenuation and spotty calcification) is evident on CT angiography **(D, E)**. Use of ^68^Ga-DOTATATE PET **(F, H, I)** clearly detected intense inflammation in this high-risk atherosclerotic plaque/distal portion of the stented culprit lesion **(arrow)** and recently infarcted myocardium (*). In contrast, using [^18^F]FDG PET **(G, J)**, myocardial spillover completely obscures the coronary arteries. CT = computed tomography; [^18^F]FDG = fluorine-18-labeled fluorodeoxyglucose; ^68^Ga-DOTATATE = gallium-68-labeled DOTATATE; PET = positron emission tomography.

**Table 1:** Baseline Clinical Factors

	Stable CVD (n = 18)	Unstable CVD∗ (n = 24)	All (n = 42)
Age, yrs	67 ± 10	71 ± 7	69 ± 9
Male	14 (78)	20 (83)	34 (81)
Body mass index, kg/m^2^	29 ± 5	27 ± 4	28 ± 5
Heart rate, beats/min	57 ± 9	58 ± 6	57 ± 8
Systolic blood pressure, mm Hg	141 ± 22	144 ± 24	143 ± 21
Diastolic blood pressure, mm Hg	74 ± 9	76 ± 10	75 ± 9
Occurrences of previous cardiovascular history			
Angina	8 (44)	4 (17)	12 (29)
Myocardial infarction	3 (17)	9 (38)	12 (29)
Coronary stenting	5 (28)	3 (13)	8 (19)
Coronary artery bypass surgery	1 (6)	3 (13)	4 (10)
Transient ischemia attack or stroke	4 (22)	3 (13)	7 (17)
Carotid endarterectomy surgery	2 (11)	0 (0)	2 (5)
Occurrences of cardiovascular risk factors			
Hypertension	9 (50)	16 (67)	25 (60)
Hypercholesterolemia	17 (94)	17 (71)	34 (81)
Noninsulin dependent diabetes	2 (11)	6 (25)	8 (19)
Smoking habit (ex or current)	10 (56)	18 (75)	28 (67)
Family history of coronary heart disease†	7 (39)	12 (50)	19 (45)
Occurrences of cardiovascular medications			
Aspirin	15 (83)	15 (63)	30 (71)
Clopidogrel	6 (33)	21 (88)	27 (64)
Statin	16 (89)	22 (92)	38 (91)
β-Adrenergic receptor blocker	11 (61)	14 (58)	25 (60)
Angiotensin converting enzyme inhibitor/receptor blocker	8 (44)	17 (71)	25 (60)
Calcium-channel blocker	3 (17)	9 (38)	12 (29)
Other antihypertensive	3 (17)	4 (17)	7 (17)
Oral nitrates	4 (22)	3 (13)	7 (17)
Random lipid profile			
Total cholesterol, mmol/l	4.0 ± 1.1	3.6 ± 0.9	3.8 ± 0.8
HDL cholesterol, mmol/l	1.1 ± 0.2	1.1 ± 0.3	1.1 ± 0.2
LDL cholesterol, mmol/l	2.1 ± 0.6	1.9 ± 0.7	2.0 ± 0.7
Triglycerides, mmol/l	1.8 ± 0.9	1.3 ± 0.5	1.5 ± 0.7
HDL cholesterol, mmol/l	3.8 ± 1.0	3.4 ± 0.7	3.6 ± 0.9
Median high-sensitivity CRP, mg/l	2.5 (0.8–3.7)	2.1 (0.7–5.8)	2.4 (0.7–4.6)
Median peak serum troponin-I concentration, ng/l‡	–	573 (59.5–3,957)	–
Median %10-year Framingham risk score	9 (8–21)	18 (11–26)	16 (8–26)
Median coronary artery calcium score, Agatston units	177 (96–680)	756 (255–1,419)	433 (120–1,314)

Values are mean ± SD, n (%), or mean (interquartile range).

ACS = acute coronary syndrome; CRP = C-reactive protein; CVD = cardiovascular disease; HDL = high-density lipoprotein; LDL = low-density lipoprotein; TIA = transient ischemic attack.

∗ Unstable CVD = ACS or TIA/stroke within the previous 3 months.

† <65 years of age.

‡ ACS patients.
