# Supplementary material for: Detection of Atherosclerotic Inflammation by 68Ga-DOTATATE PET Compared to [18F]FDG PET Imaging
Source: J Am Coll Cardiol. 2017 Apr 11;69(14):1774–91. doi: 10.1016/j.jacc.2017.01.060 (PMC5381358; doi:10.1016/j.jacc.2017.01.060)

**ONLINE APPENDIX**

**EXTENDED METHODS**

**EXCLUSION CRITERIA**: renal impairment (eGFR<30mL/min), history of contrast nephropathy or contrast allergy, systemic vasculitis or other active inflammatory disease, atrial fibrillation, hemorrhagic stroke, women of childbearing potential, and inability to lie flat during scanning.

**PET-CT IMAGING.** Imaging was performed during two visits. Visit one: patients underwent electrocardiography (ECG)-gated PET-CT imaging with either ^68^Ga-DOTATATE or ^18^F-fluorodexyglucose (FDG). Visit two: PET-CT imaging was repeated using the alternate tracer, with at least 24 hours between imaging sessions to allow full radiotracer decay. The order of scans was determined by practical reasons, namely patient and scanner availability. PET-CT imaging was completed prior to carotid endarterectomy in patients scheduled for surgery, but did not delay the timing of surgery.

At the start of imaging, patients were positioned supine, headfirst on the PET-CT scanner with their arms rested comfortably above their heads. After antero-posterior and lateral scouts, a low-dose attenuation correction CT was acquired followed by an unenhanced CT (120kV, smart mA) for anatomical co-registration. The first six patients underwent 90-minute dynamic ^68^Ga-DOTATATE PET imaging in one bed position, covering the heart, with venous blood sampling to provide an estimate of the plasma-to-whole blood ratio time course, which was combined with an image-derived arterial whole blood time-activity curve, to produce an input function for kinetic modeling.

The plasma concentration of ^68^Ga-DOTATATE vs. time from patients who underwent dynamic imaging showed that following an initial rapid redistribution phase, the radiolabel displayed first order exponential decay, with a plasma half-life of 8.6 ± SD 1.5 minutes. Volume of distribution was 4.1 ± SD 0.7 L, consistent with a blood compartment, and clearance was rapid, 361.6 ± SD 110.7 ml/minute. Logan plot analysis indicated that the tissue signal equilibrium had occurred by 40 minutes after ^68^Ga-DOTATATE injection, and static scanning therefore commenced at this start point for all subsequent imaging.

Static ^68^Ga-DOTATATE PET scans were first performed covering the heart and aorta from the superior margin of the aortic arch to its bifurcation, for six minutes per bed position. After 60 minutes from time of injection, ^68^Ga-DOTATATE carotid PET images were acquired for 20 minutes with the patient remaining in a supine position, arms secured at their sides, and head positioned in a neck holder to minimize movement. Based on previous work, a 90-minute circulation time was adopted for ^18^F-FDG scans, with four-minute image acquisition per cardiac and aortic bed position, and 15 minutes for the carotids.

ECG-gated CT calcium scoring and CTCA were performed during one of the two visits, prior to PET imaging, using either the combined GE Discovery 690 PET-CT scanner with integrated 64-slice CT scanner or a 128-slice dual-source Siemens SOMATOM Definition Flash scanner (Siemens, Germany). For improved plaque characterization, patients with a primary diagnosis of coronary atherosclerosis, representing roughly half the total patient population, underwent CTCA imaging using the 128-slice scanner. CT imaging was performed as per standard clinical protocols. Coronary calcium scans were acquired with a 3 mm slice thickness and reconstructed with the B35f kernel. Sublingual GTN (400 mcg, two sprays) was administered prior to CTCA, and intravenous metoprolol (2.5 to 20 mg) given as required to achieve a target heart rate of <60bpm. CTCA was acquired with either prospective or retrospective triggering depending on resting heart rate, whole-volume single-breath hold acquisition, with tube current and voltage (100 or 120 kV) adjusted for body size, pitch 3.2, rotation time 0.28 seconds, temporal resolution 75 milliseconds, and isotropic and cross plane resolution 0.33 mm. Following a test bolus to determine the delay in seconds from time of contrast injection to image acquisition, intravenous contrast (70mL NIOPAM 350, Bracco, UK) was administered at 6 mL/second, followed by 40mL 0.9% saline flush. Slice thickness was 0.75 mm and B26f kernel applied.

CT carotid angiography was performed after PET scanning on one of the two visits (alternate visit to coronary angiography), with the patient unmoved, using the combined PET-CT scanner. Contrast-enhanced CT images were acquired from the aortic arch to the circle of Willis, using bolus tracking (trigger at 100 HU above baseline) with an ROI placed in the aortic arch, and 100mL NIOPAM 350 at 5mL/second followed by a 50mL flush of 0.9% saline flush (max 200 mA, 120kV), slice thickness 0.625mm), and reconstructed using H20f kernel. The maximum total estimated radiation exposure per patient was 30 mSv.

**IMAGE ANALYSIS.** PET images acquired in 3.27 mm slice thickness (2.74 x 2.74 x 3.27 mm voxel size) were reconstructed using 3D iterative time-of-flight ordered-subset expectation maximization (three iterations, 24 subsets) with point spread function modeling to reduce partial volume error, a 2.0 mm post-filter, and corrections applied for attenuation, dead time, decay, normalization, random coincidences and scatter. As per similar studies,(1, 2) respiratory motion correction was not attempted; this correction remains experimental for coronary PET imaging.

Using CT angiography as the anatomical reference standard, 2D regions of interest (ROIs) were drawn around outer arterial wall boundaries on consecutive fused PET-CT slices, re-sampled to 2.99 mm and oriented in the axial plane, with orthogonal views used to confirm accuracy of PET-CT co-registration performed using the automatic function in Osirix, and to verify anatomic signal location in three planes. To avoid potential errors in coronary PET-CT registration, PET images were not fused to CT angiographic images for purpose of signal quantification, as roughly half of the patients had CT coronary angiogram performed using a separate scanner.

For each patient, ROIs were drawn: (i) around all major (diameter >2 mm) epicardial coronary arteries divided into segments according to American Heart Association classification; (ii) three slices (0.9 mm) below the carotid bifurcation to ten slices (3 cm) above; and (iii) covering the thoracic aorta. ROIs were extended above or below the pre-determined carotid reference segment in patients with symptomatic carotid disease as needed to capture the entire diseased region, with an equal number of ROIs on each side. Efforts were made to avoid ROIs significantly affected by spillover from surrounding structures; for this reason, distal right coronary segments in close proximity to high physiological liver signals were excluded from analysis. The superior aspect of the aortic arch was also not analyzed due to the high noise level arising from its location at the axial edge of the scanner field of view.

In total, 4986 ^68^Ga-DOTATATE and 4525 ^18^F-FDG ROIs were drawn around 230 coronary segments, 70 carotid arteries, and 42 aortas. Within each 2D ROI, maximum voxel value was derived to estimate maximum tissue-to-blood ratio (TBR_max_), normalized by mean blood pool activity measured within five consecutive circular ROIs drawn within the lumen of the superior vena cava. This was aimed at sampling the highest inflammatory signal, although maximal voxel values are subject to noise. Consequently, TBR_max_ values from neighboring slices were averaged to produce “mean of the whole vessel” (m), and “most diseased segment” (mds), defined as the mean of three consecutive ROIs centered on the single hottest slice. For atherosclerosis imaging, TBR is recommended over standardized uptake value (SUV) as there is better normalization for tracer delivery, errors in injected activity do not propagate into TBR values, and the results are more stable as a function of the imaging time-point.(3) TBR also demonstrates closer association with histological markers of inflammation than SUV.(4) Excellent intra- and inter-observer agreement has been shown previously using these methods for carotid and aortic ^18^F-FDG imaging.(5) In this study, reproducibility of ^68^Ga-DOTATATE TBR measurement was tested by 2 independent observers using 10% of the coronary and carotid scans (n=4 for both), selected at random, with 1 week in between intra-observer reads. The predefined threshold for evaluation of coronary PET images was myocardial maximum SUV<5.0 in the basal ventricular septum.(6) Coronary calcium scoring was calculated as per the Agatston method.

**RNA-SEQUENCING.** RNA sequencing data (ribodepleted) were downloaded from EGA (http://dcc.blueprint-epigenome.eu/#/datasets) and processed as follows: Trim Galore 0.3.7(http://www.bioinformatics.babraham.ac.uk/projects/trim_galore/) with parameters “-q 15 -s 3 --length 30 -e 0.05” used to remove PCR and sequencing adapters off. Trimmed reads were then aligned to the Ensembl v70 human transcriptome using Bowtie 1.0.1 (PMID: 19261174) with parameters “-a --best --strata -S -m 100 -X 500 --chunkmbs 256 --nofw --fr”. MMSEQ 1.0.8a with default parameters (PMID: 24281695, 21310039) used to quantify gene expression. MMDiff with parameters “-fixalpha -p 0 -uhfrac 1.0” was used to summarize and normalize gene expression for cell type.

**QUANTITATIVE PCR.** Peripheral blood mononuclear cells were isolated by Percoll gradient centrifugation, and monocytes separated by CD3 depletion using MACS (Miltenyi Biotec, Germany). Cells were then cultured in M-CSF 50 ng/mL for seven days prior to stimulation with lipopolysaccharide 100 ng/mL for 18 hours. Macrophage isolation was confirmed by flow cytometry (CD14+/CD11b+) and immunostaining. Macrophage RNA was extracted using the RNeasy kit (Qiagen, Germany), and strand cDNA synthesis carried out using Superscript III reverse Transcriptase (Invitrogen, USA). qPCR was performed using Rotor-gene SYBR green in a RotorGene thermocycler (Corbett Life Science, Australia) with the following primers: *SSTR2* forward (GCCAAGATGAAGACCATCAC) and reverse (GATGAACCCTGTGTACCAAGC); *HPRT* forward (CAGGACTGAACGTCTTGCTC) and reverse (CAAATCCAACAAAGTCTGGC). *SSTR2* gene expression was normalized to the expression of *HPRT*, and displayed as fold-change using the comparative Ct method (2^-ΔΔCt^).

Using similar methods, *SSTR2* and *CD68* qPCR was also performed on carotid tissue sections taken from a 0.9 cm block extending below the level of the bifurcation. This orientation was consistent across all specimens and selected for ease of comparison with clinical imaging. The following additional primers were used: *CD68* forward GCAGCACAGTGGACATTCTC; reverse GGTGGACAGCTGGTGAAAGA. Gene expression was normalized to the expression of *HPRT*; normalized gene expression = 2-ΔCt (ΔCt = Ct gene of interest – Ct HPRT).(7) Gene expression in carotid plaques was compared with ^68^Ga-DOTATATE mTBR_max_ measured at the corresponding level (the carotid bifurcation and two slices below) in clinical images.

**^68^Ga-DOTATATE AUTORADIOGRAPHY.** Carotid specimens were collected at the time of surgery and stored at -70 ^o^C. Specimens were orientated using clinical images and consecutive 30 µm cryostat sections cut at 0.9 cm superior to the carotid bifurcation, then cold mounted and fixed onto microscope slides. Ligand binding was carried out as previous described.

(8) Carotid sections were pre-incubated in 300 μL HEPES (4-[2-hydroxyethyl]-1-piperazineethanesulfonic acid) 10 mM buffer for five minutes, and then placed in 300 μL ^68^Ga-DOTATATE 10 nM solution for 30 minutes at room temperature (estimated specific activity 34 GBq/μmol(9)). The tracer concentration applied was within one order of magnitude to the average plasma concentration of ^68^Ga-DOTATATE measured at the start of clinical imaging. Adjacent sections were incubated with a competing concentration of unlabeled DOTATATE (1 µM) to define regions of non-specific binding. At the end of the experiment, sections were washed in Tris-HCL (pH 7.8), dipped in distilled H_2_O and opposed to phosphor screen. Optimum exposure time was determined empirically, with radioactivity within sections visualized using a Cyclone Storage Phosphor System (PerkinElmer, USA) and OptiQuant image acquisition and analysis software.

**IMMUNOSTAINING.** Immunostaining was conducted as previously described.(10) Cryostat sections (30 µm) were fixed in 4% formaldehyde for ten minutes before incubating with a rabbit antiserum (ab9550; Abcam, UK) raised to a 21 amino acid peptide corresponding to 339-359 C-terminus residues of the somatostatin sub-type 2 (SST_2_) receptor, a region with no sequence similarity by BLAST analysis with any of the other four somatostatin receptors. Cryosections were incubated with antisera diluted to 1:100 in buffer for one hour. Binding of the primary antisera was visualized by the peroxidase-antiperoxidase technique, with counterstaining using hematoxylin. Controls included omission of the primary antisera in adjacent sections and substitution of the primary antisera with serotype-matched IgG to confirm no detectable staining. Adjacent sections were stained with mouse antisera to the pan-macrophage marker CD68 (M0876; 1:1000 dilution; Dako, Agilent Technologies, USA) using a similar protocol, and Movat’s pentachrome stain for anatomic characterization. Stained slides were visualized using light microscopy (Olympus BX51, Japan).

^68^Ga-DOTATATE autoradiography and immunostaining was also carried out on cultured macrophages as above; SST_2_ immunofluorescence was performed using 1:50 primary antisera dilution, with nuclear DAPI counterstain, and visualized with confocal microscopy (Leica TCS SP5, Germany). SST_2_ (1:25 dilution) and CD68 (1:100 dilution) confocal immunofluorescence microscopy was similarly performed on sections of carotid plaque. In parallel, isotype-matched IgG was used at the same concentrations as a negative control of both antibodies.

**REFERENCES**

**1.** Rogers IS, Nasir K, Figueroa AL, et al. Feasibility of FDG imaging of the coronary arteries. JACC: Cardiovascular Imaging 2010;3:388–397.

**2.** Joshi NV, Vesey AT, Williams MC, et al. 18F-fluoride positron emission tomography for identification of ruptured and high-risk coronary atherosclerotic plaques: a prospective clinical trial. Lancet 2014;383:705–713.

**3.** Bucerius J, Hyafil F, Verberne HJ, et al. Position paper of the Cardiovascular Committee of the European Association of Nuclear Medicine (EANM) on PET imaging of atherosclerosis. Eur J Nucl Med Mol Imaging 2016;43:780–792.

**4.** Asabella AN, Ciccone MM, Cortese F, et al. Higher reliability of 18F-FDG target background ratio compared to standardized uptake value in vulnerable carotid plaque detection: a pilot study. Ann Nucl Med 2014;28:571–579.

**5.** Rudd JHF, Myers KS, Bansilal S, et al. (18)Fluorodeoxyglucose positron emission tomography imaging of atherosclerotic plaque inflammation is highly reproducible: implications for atherosclerosis therapy trials. J Am Coll Cardiol 2007;50:892–896.

**6.** Wykrzykowska J, Lehman S, Williams G, et al. Imaging of inflamed and vulnerable plaque in coronary arteries with 18F-FDG PET/CT in patients with suppression of myocardial uptake using a low-carbohydrate, high-fat preparation. J Nucl Med 2009;50:563–568.

**7.** Jager NA, Wallis de Vries BM, Hillebrands JL, et al. Distribution of matrix metalloproteinases in human atherosclerotic carotid plaques and their production by smooth muscle cells and macrophage subsets. Mol Imaging Biol 2016;18:283–291.

**8.** Irkle A, Vesey AT, Lewis DY, et al. Identifying active vascular microcalcification by 18F-sodium fluoride positron emission tomography. Nat Commun 2015;6:1–11.

**9.** Poeppel TD, Binse I, Petersenn S, et al. 68Ga-DOTATOC Versus 68Ga-DOTATATE PET/CT in Functional Imaging of Neuroendocrine Tumors. J Nucl Med 2011;52:1864–1870.

**10.** Davenport AP, Kuc RE. Cellular localization of receptors using antibodies visualized by light and dual labeling confocal microscopy. Receptor Binding Techniques 2012;897:239–260.

**ONLINE FIGURES & TABLES**

**
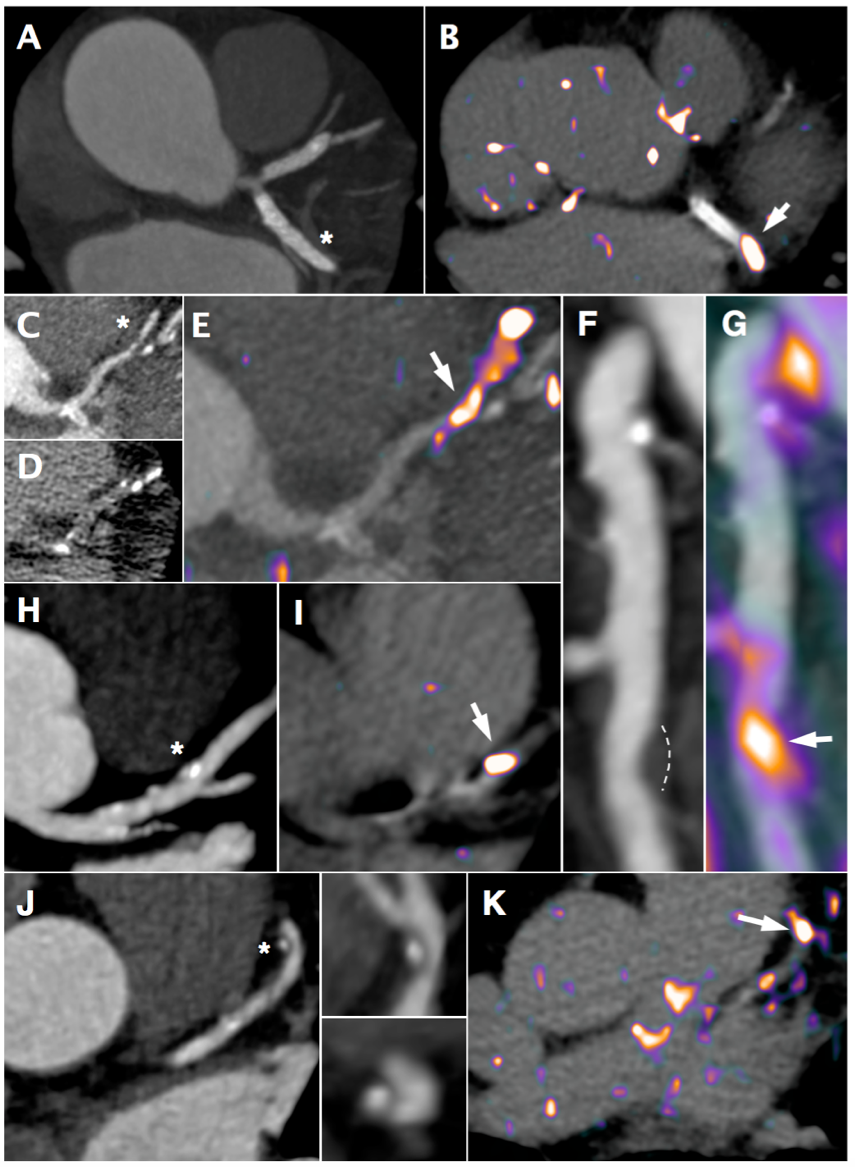
**

**ONLINE FIGURE 1 Coronary ^68^Ga-DOTATATE PET inflammation imaging**

Five representative cases of coronary inflammation imaging with ^68^Ga-DOTATATE PET.

Case 1: CT angiogram (**A**) showing a culprit left circumflex ACS lesion (*****), with increased inflammation in the distal stented artery (arrow) detected by ^68^Ga-DOTATATE PET (**B**).

Case 2: CT angiogram (**C**) and CT calcium scan (**D**) from a patient with stable coronary disease, with increased inflammation (arrow) detected by ^68^Ga-DOTATATE PET (**E**) in the mid left anterior descending coronary artery lesion (*), and lack of signal in the first diagonal artery containing increased calcification.

Case 3: CT angiogram (**F**) and ^68^Ga-DOTATATE PET (**G**) images showing increased inflammation in a non-calcified stable mid right coronary artery lesion (arrow), with positive remodeling (dashed line).

Case 4: CT angiogram (**H)** and ^68^Ga-DOTATATE PET (**I**) images showing inflammation in a left anterior descending artery plaque (arrow) with spotty calcification (*****).

Case 5: CT angiogram (**J**) and ^68^Ga-DOTATATE PET (**K**) images showing inflammation in a non-calcified stable left anterior descending artery lesion (arrow) with low attenuation (*).


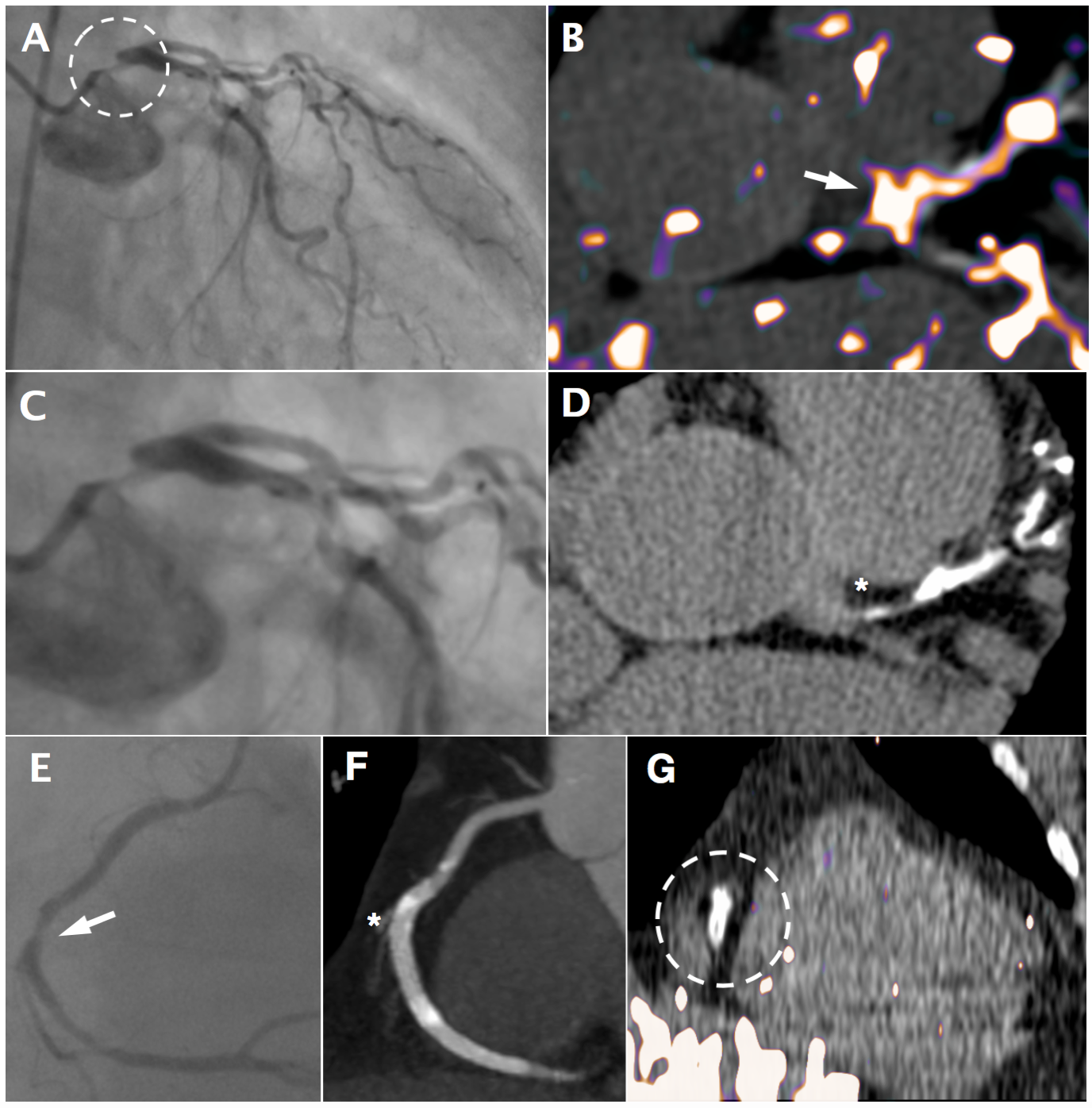


**ONLINE FIGURE 2 Native culprit ACS lesion and stable stented coronary lesion imaged using ^68^Ga-DOTATATE PET**

Coronary angiogram (**A**, **C**) from an 81-year old woman with acute coronary syndrome resulting from a severe left main stem stenosis (hatched oval); intense inflammation arising from this native culprit lesion (arrow, *) detected using ^68^Ga-DOTATATE PET (**B**), with corresponding CT image (**D**). In contrast, stable coronary lesion (arrow) in a 55-year old man shown on x-ray angiography (**E**), with CT angiography (**F**) and ^68^Ga-DOTATATE PET (**G**) performed two weeks after percutaneous coronary stenting (*****). Note absence of inflammatory signal arising from the recently stented stable coronary lesion (hatched oval).

**
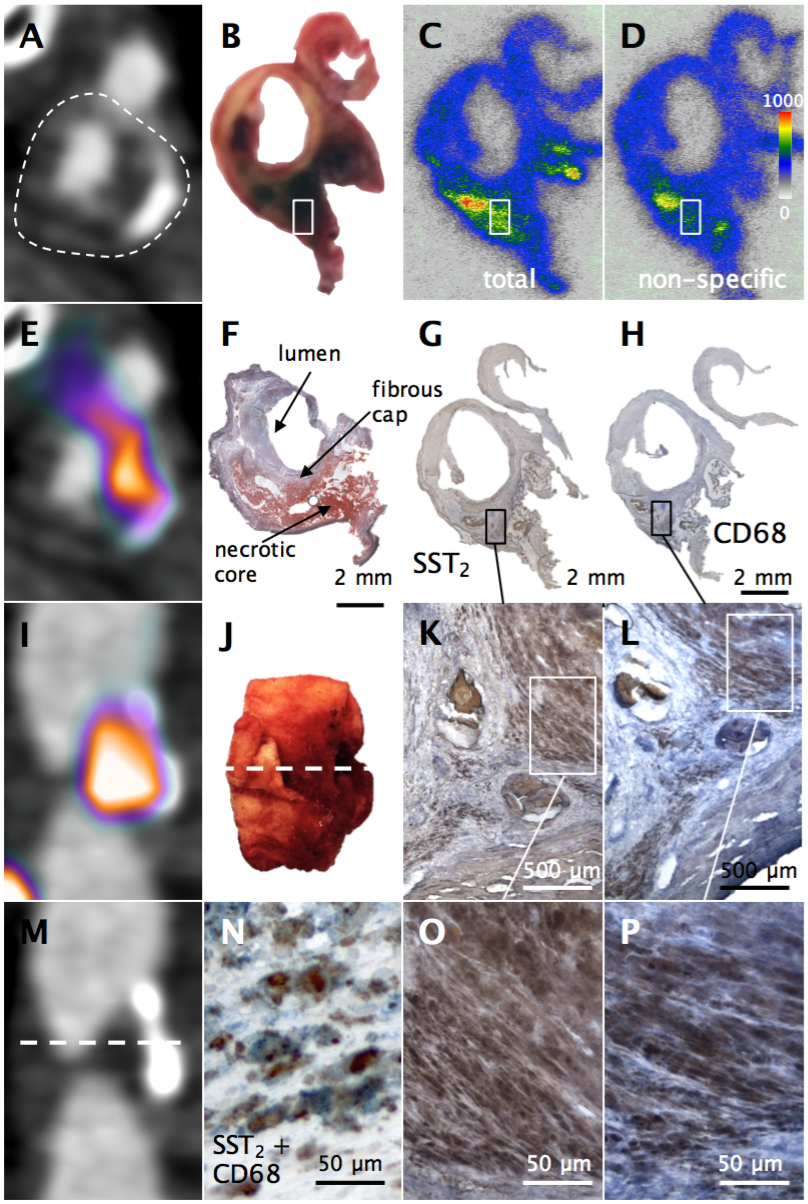
**

**ONLINE FIGURE 3 ^68^Ga-DOTATATE Ligand Binding to Macrophage SST_2_ in Carotid Plaque #2**

*In vivo*: CT angiography of a culprit internal carotid artery (hatched oval) in axial (**A**) and coronal (**M**) views, with corresponding fused ^68^Ga-DOTATATE PET-CT images (**E, I**). *Ex vivo*: macrographic image of the explanted carotid specimen (**B, J**); phosphor autoradiographic image showing the total binding of ^68^Ga-DOTATATE to SST_2_ receptors in macrophages (**C**), within a transverse carotid section corresponding to the level shown in clinical images (hatched line). Adjacent slide incubated with ^68^Ga-DOTATATE and cold competing ligand (**D**), showing very low levels of non-specific binding. Bright-field photomicrographs showing brown immunoreactive SST_2_ staining (**G, K, O**) of macrophages identified with the pan-macrophage marker CD68 (**H, L, P**); co-localized SST_2_ (brown) and CD68 (blue) staining in the same section (**N**); Movat’s pentachrome stain (**F**).

**ONLINE TABLE 1 Clinical predictors of inflammatory PET signals**

Clinical factors showing statistical significance (p<0.05) for prediction of vascular PET mTBR_max_ values on univariate linear regression were evaluated together using multivariate regression; significant factors determined by multivariate regression highlighted in bold, with total adjusted R^2^ values shown in the far-right column.


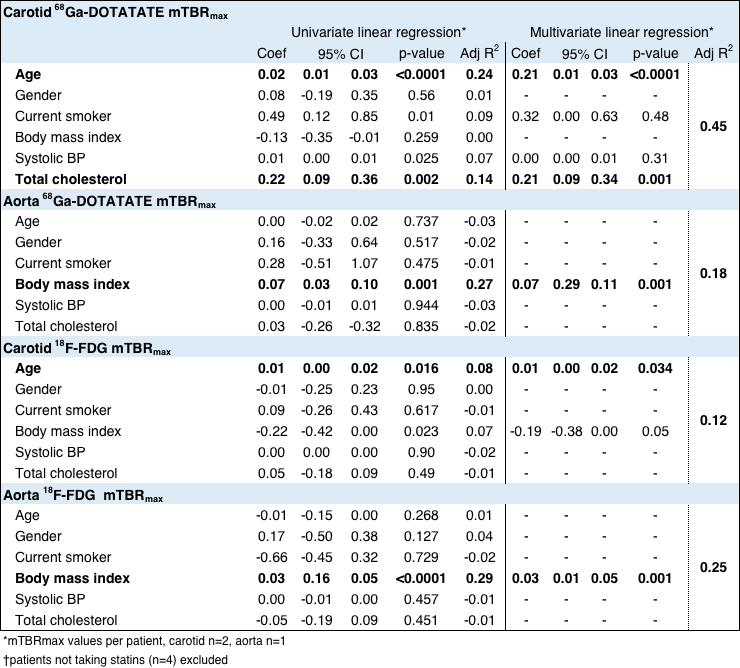

Supplement: Online Data [file mmc1.docx]
